# Multimodal analysis reveals asynchronous aging dynamics between endometriosis-associated ovarian cancer and endometriosis patients

**DOI:** 10.1186/s12916-026-05101-6

**Published:** 2026-07-27

**Authors:** Pawel Kordowitzki, Kejun Ying, Maximilian H. Beck, Renata Voltolini Velho, Charlotte Engelbrecht, Jalid Sehouli, Sylvia Mechsner

**Affiliations:** 1 Department of Basic and Preclinical Sciences, Nicolaus Copernicus University, Torun, Poland; 2 Department of Gynecology with Center for Oncological Surgery, Campus Virchow Klinikum, Charité-Universitätsmedizin Berlin, Berlin, Germany; 3 Institute of Advanced Studies, Nicolaus Copernicus University, Torun, Poland; 4 T. H. Chan School of Public Health, Harvard University, Boston, MA USA; 5 Wu Tsai Neurosciences Institute, Stanford University, Stanford, CA USA

**Keywords:** endometriosis-associated ovarian cancer, DNA methylation, biological age, longevity marker, machine learning models, telomeres, aging

## Abstract

**Background:**

The malignant transformation of endometriosis into endometriosis-associated ovarian cancer (EAOC) is still of great interest among researchers. Despite the shared inflammatory and hormonal drivers, the cellular aging dynamics differentiating these two pathologies remain elusive. This study aimed to quantify biological aging using DNA methylation status and telomere length to identify distinct signatures associated with disease progression.

**Methods:**

We conducted a comparative analysis of multiple aging-related biomarkers in blood, including biological age, telomere length, and DNA methylation status, as well as specific longevity markers, across cohorts of age-matched patients with endometriosis and EAOC, compared with unaffected controls. Precision assessment utilized machine learning models to determine biological age deviation and methylation levels at specific CpG sites. Finally, the study employs a Random Forest machine learning classifier to assess the discriminative power of gene signatures, complemented by Gene Ontology enrichment.

**Results:**

Our analysis revealed a profound divergence in biological aging trajectories. Patients with endometriosis exhibited significant age acceleration (+ 16.92%) and maintained telomeres that were significantly longer (13.18 ± 0.46 kbp). Conversely, EAOC patients displayed a biologically younger profile compared to their chronological age (− 14.64%). These patients also demonstrated significant telomeric attrition (12.45 ± 0.72 kbp; *p* < 0.001), reflecting their extensive mitotic history and the rapid turnover of malignant cells. Age-related methylation was the most robust marker, distinguishing the groups with high statistical significance.

**Conclusions:**

This study provides novel evidence that the transition from endometriosis to EAOC involves a fundamental shift in biological age and in telomere dynamics. Identifying these divergent aging signatures offers a powerful tool for precision oncology, enabling the development of predictive models to identify patients at high risk of malignant transformation and facilitating personalized interceptive strategies based on biological, rather than chronological age.

**Supplementary Information:**

The online version contains supplementary material available at https://doi.org/10.1186/s12916-026-05101-6.

## Background

Globally, aging is one of the primary risk factors for numerous cancers, with epithelial ovarian cancer incidence rising significantly with advancing age, and prognosis often worsening in elderly patients [1–4]. This age-related increase in ovarian cancer is intricately linked to the complex biological processes of aging, including cellular senescence and alterations in ovarian physiology [5, 6]. The accumulation of senescent cells, epigenetic alterations, genomic instability, and a pro-inflammatory microenvironment creates a permissive landscape for tumorigenesis in the aging ovary [7–9]. Previous studies have demonstrated an increased risk of ovarian cancer among women with a general history of endometriosis, with pooled relative risk estimates of approximately 1.93, particularly [10–14]. This malignant transformation, though rare (0.5–1% of endometriosis cases), represents a critical clinical concern [15, 16]. This pathological association is characteristically confined to endometrioid carcinoma and clear cell carcinoma, which constitute discrete histological subtypes within the endometriosis-associated ovarian cancer (EAOC) type. Additionally, oxidative stress stemming from increased free iron in endometriotic cysts, chronic inflammation, and hormonal imbalances, particularly hyperestrogenism, is recognized as a driver of malignant progression [15, 17, 18]. However, identifying reliable early markers for malignant transformation remains a significant challenge for clinicians and scientists. There is no doubt that the pathogenesis of EAOC is multifactorial, involving a complex interplay of genetic, molecular, and epigenetic factors, which is of great importance, especially in patients with endometriomas [19–22]. Notably, EAOC has been described as arising predominantly from endometriotic lesions rather than from the ovarian surface epithelium itself in women of different ages. In this context, it is worth mentioning that chronological age, while a primary risk factor for numerous cancers, fails to account for the substantial heterogeneity in physiological aging among individuals [23]. Biological age estimations, particularly through epigenetic clocks, offer a promising avenue for understanding how aging influences cancer outcomes and for identifying individuals at higher risk of adverse effects from cancer therapies. Importantly, emerging causality-informed epigenetic clocks, such as DamAge, distinguish between accumulated molecular damage and adaptive responses, offering superior predictive power for morbidity over chronological metrics [24, 25]. These advanced measures of biological aging, derived from DNA methylation patterns, have demonstrated significant associations with both cancer risk and survival, indicating their potential as prognostic indicators [26, 27]. Specifically, the differential between predicted methylation age (m-age) and chronological age has been identified as a critical factor in understanding cancer development and survival, with various epigenetic clocks, such as those developed by Hannum and Horvath, widely applied across diverse tissues and cell types [28–36]. Regional hypermethylation targeted at specific CpG-rich promoter regions leads to the epigenetic silencing of critical loci involved in tumor suppression, immunological regulation, and cell cycle orchestration. These predictable patterns have facilitated the development of “epigenetic clocks,” which utilize the methylation status of distinct CpG sites to estimate biological age. Therefore, the aim of this study was to elucidate the role of aging and DNA methylation in endometriosis-associated ovarian cancer and to identify novel biomarkers for this specific cancer. Moreover, this study aims to clarify whether biological age and longevity markers differ between endometriosis and EAOC, thereby better understanding the malignant transformation of endometriosis into cancer.

## Methods

### Ethical approval 

The study was approved by the ethics commissions of Charité – Universitätsmedizin Berlin (EA2/266/22 and EA1/150/24). Blood samples from patients were obtained with informed consent, following local institutional review and the Declaration of Helsinki. 

### Study design

**Fig. 1:**
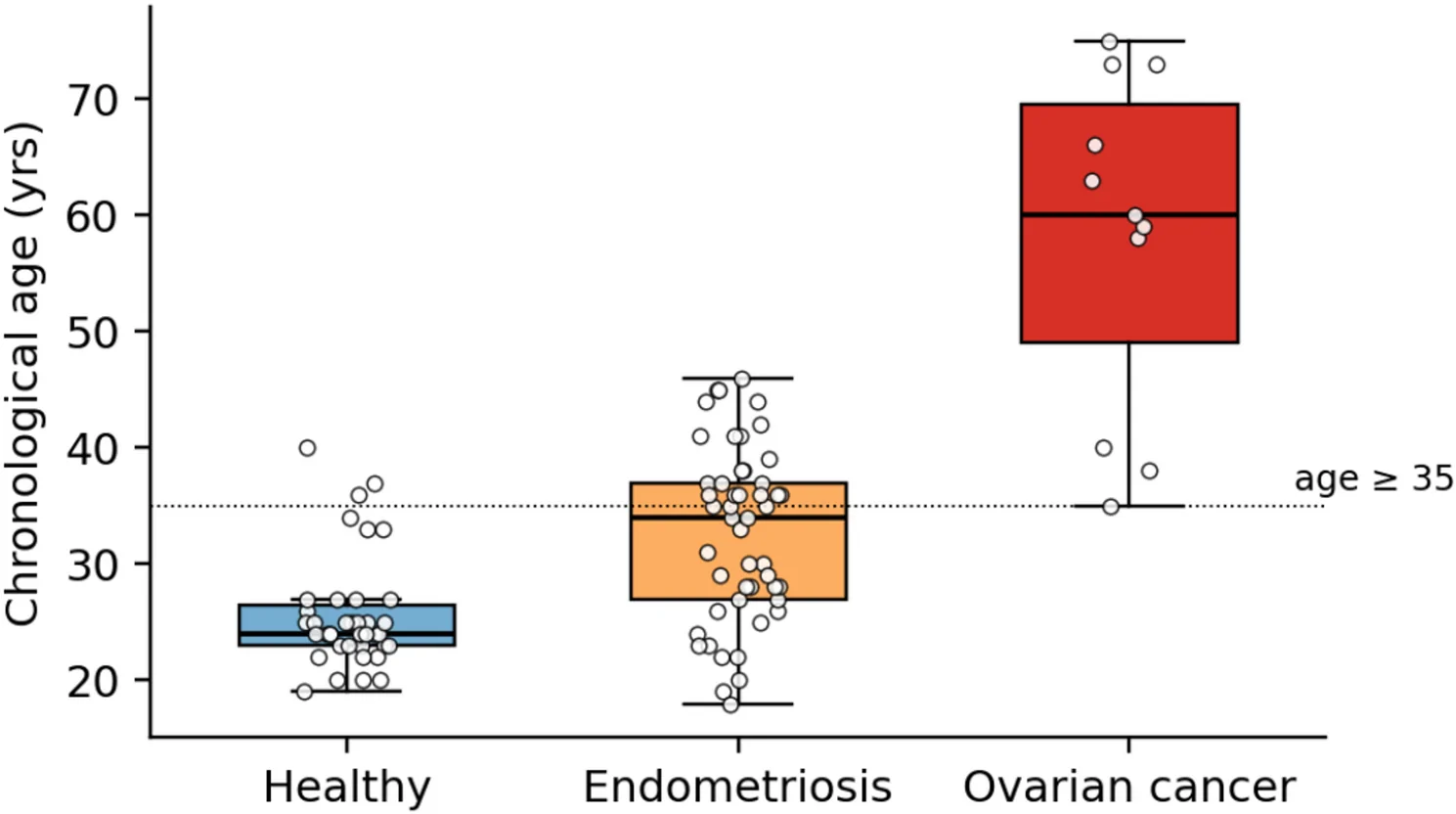
Chronological age distribution by group. Healthy *n* = 39, median: 24 years (19–40). Endometriosis *n* = 49, median: 34 (18–46). Ovarian cancer *n* = 11, median: 60 (35–75). Only the age ≥ 35 stratum supports matched comparison

This study evaluated patients with histologically confirmed endometriosis-associated ovarian cancer at the time of primary treatment (n = 11, age range 35–75 years), endometriosis patients (n = 49, age range 18–45 years), and unaffected healthy individuals (n = 39, age range 19–40 years). In the endometriosis cohort, all patients with a history of endometriosis were included without separating by type or grade. The chronological age distribution of the cohorts is shown in Fig. 1. The study was conducted at Charité – Universitätsmedizin Berlin, a German tertiary academic medical center and certified European Society of Gynecological Oncology (ESGO) Center of Excellence. Eligible were patients who received primary treatment for an endometriosis-associated ovarian tumor and for endometriosis at the Department of Gynecology of the Charité – Universitätsmedizin Berlin between January 1, 2014, and December 31, 2025. Exclusion criteria included primary surgery performed at another institution, ovarian metastases from non-ovarian malignancies, or incomplete clinicopathologic or follow-up data. There were no restrictions regarding treatment strategies. Patients were identified through the institutional cancer registry. The healthy individuals in the control group agreed to voluntarily participate in this study. After sampling, the blood was kept frozen at -80 degree until analysis. Clinical and pathological data were extracted from electronic medical records and the institutional pathology database.

### Biological age marker analysis

Raw values analyzed after stripping unit suffixes (%, kbp). Three-group Kruskal–Wallis, pairwise two-sided Mann–Whitney U, Cliff’s δ with 2000-iteration bootstrap 95% CI, Benjamini–Hochberg correction across 7 markers. Age adjustments: healthy-only, pooled, ANCOVA. Primary test: pairwise MWU on age ≥ 35 subset. Classifier: nested 5-fold stratified CV with logistic regression (age-only baseline) and elastic-net (age-adjusted panel). Python 3.14: pandas, scipy.stats, statsmodels, scikit-learn.

### Identification of differentially expressed genes based on the Turku endometriosis database

For the Turku Endometriosis Database, endometrial tissue samples were obtained from patients diagnosed with endometriosis (*n* = 345) and age-matched healthy controls (*n* = 63), following informed consent and institutional review board approval [37]. A more detailed cohort description, including the inclusion and exclusion criteria of the aforementioned database, can be found in the respective reference [37]. For the latter-mentioned database, total RNA was extracted from tissue samples and subjected to microarray-based gene expression profiling. Gene expression levels were quantified and normalized across samples. Quality control measures were applied to remove low-quality samples and genes with insufficient detection rates. A targeted panel of 215 genes (see supplementary file 1) was analyzed, including genes previously implicated in endometriosis pathogenesis, cell cycle regulation, DNA repair, epigenetic modification, and reproductive function. To identify genes whose expression levels differ between endometriosis patients and healthy controls, we performed differential expression analysis using Welch’s t-test for each gene. This method was chosen to account for unequal variances and sample sizes between groups. Prior to differential expression analysis, raw gene expression values were normalized to reduce technical variability and improve comparability across samples. For each gene, we calculated: (1) mean expression in diseased and healthy groups, (2) log₂ fold change (log₂FC) defined as log₂[(diseased mean + 0.001)/(healthy mean + 0.001)], (3) t-statistic and p-value using two-tailed Welch’s t-test, and (4) effect size (Cohen’s d) to quantify the magnitude of difference. Multiple testing correction was applied using the Benjamini-Hochberg false discovery rate (FDR) method, with significance defined as FDR < 0.05. A total of 73 genes were identified as significantly differentially expressed (31 upregulated, 42 downregulated in endometriosis).

### Gene ontology enrichment analysis

Significantly differentially expressed genes (*n* = 73, FDR < 0.05) were subjected to gene ontology (GO) enrichment analysis to identify overrepresented biological processes, molecular functions, and cellular components. GO enrichment was performed using online tools such as g: Profiler (https://biit.cs.ut.ee/gprofiler/), DAVID (https://david.ncifcrf.gov/), or Enrichr (https://maayanlab.cloud/Enrichr/) with the following parameters: organism = Homo sapiens, sources = GO Biological Process (GO: BP), significance threshold method = FDR, user threshold = 0.05. GO terms with a minimum of 2 genes and FDR < 0.05 are considered significantly enriched. The rationale for selecting the top 50 genes was based on combined ranking according to differential expression magnitude, biological relevance, and statistical significance to facilitate downstream pathway analyses while reducing noise from weaker associations.

### Biomarker identification

Candidate biomarker genes for endometriosis were prioritized using a multidimensional evaluation framework designed to integrate statistical significance with biological and clinical relevance. Five principal criteria were considered:

*(1) Statistical Robustness*.

Genes were initially ranked according to the strength and reliability of differential expression between endometriosis and healthy control samples. This assessment included the magnitude of differential expression (log₂ fold change), statistical significance (Welch’s t-test p-value), multiple-testing adjusted significance (false discovery rate, FDR), and effect size (Cohen’s d). Preference was given to genes exhibiting large effect sizes (Cohen’s d ≥ 0.8), substantial expression differences (|log₂FC| ≥ 1), and statistically significant results after Benjamini–Hochberg correction (FDR < 0.05). Genes demonstrating consistent expression differences with low within-group variability were considered particularly robust candidates.

*(2) Biological Relevance to Endometriosis Pathophysiology*.

Genes were evaluated for their known or plausible involvement in biological processes implicated in endometriosis development and progression. Particular emphasis was placed on genes associated with chronic inflammation, immune dysregulation, oxidative stress, extracellular matrix remodeling, angiogenesis, steroid hormone signaling, cellular proliferation, apoptosis, DNA damage response, epigenetic regulation, cellular senescence, and reproductive function. Functional annotations were assessed using pathway enrichment analyses and a comprehensive review of the existing literature.

*(3) Discriminative Power.*

The ability of individual genes to distinguish endometriosis patients from healthy controls was evaluated as a measure of diagnostic potential. Genes demonstrating clear separation between disease and control groups, minimal overlap in expression distributions, and high classification performance were prioritized. When applicable, receiver operating characteristic (ROC) analyses and area under the curve (AUC) metrics were used to quantify discriminative capacity, with higher AUC values indicating superior diagnostic performance.

*(4) Clinical Applicability.*

Genes were further assessed according to their potential translational value. Preference was given to biomarkers that could be measured using clinically feasible assays and that possess potential utility for diagnosis, disease monitoring, risk stratification, or therapeutic intervention. Additional consideration was given to genes encoding proteins involved in pharmacologically targetable pathways or molecular mechanisms relevant to disease progression and treatment response.

*(5) Reproducibility Across Independent Datasets.*

To enhance reliability and reduce the likelihood of false-positive findings, candidate biomarkers were evaluated for consistency across independent cohorts, publicly available transcriptomic datasets, and previously published studies whenever available. Genes exhibiting concordant expression patterns across multiple datasets and analytical platforms were considered more reproducible and therefore more likely to represent biologically meaningful markers of endometriosis.

Genes fulfilling multiple criteria simultaneously were considered the strongest biomarker candidates and were selected for subsequent downstream analyses and validation. We acknowledge that some degree of exploratory interpretation remains inherent to biomarker prioritization.

### Predictive modeling

To assess the utility of differentially expressed genes for classifying endometriosis samples, we constructed machine learning models using the top 50 most significantly differentially expressed genes as features. A Random Forest classifier (n_estimators = 100, max_depth = 10, random_state = 42) was trained using an 80/20 train-test split with stratified sampling to maintain class balance. Features were standardized using z-score normalization (StandardScaler) prior to model training. Model performance was evaluated using standard classification metrics including accuracy, area under the receiver operating characteristic curve (AUC-ROC), confusion matrix, and 5-fold cross-validation. The final model achieved 85.4% accuracy and 83.7% AUC on the held-out test set, demonstrating strong discriminative power of the gene expression signature. AUC values should be interpreted as preliminary and exploratory due to the absence of an independent validation cohort. We emphasize that future validation studies in larger external cohorts are required.

### Data visualization

All visualizations were generated using Python 3.10 with matplotlib (v3.8.0), seaborn (v0.13.0), and scikit-learn (v1.3.0). Sample distribution was visualized as bar plots and pie charts. Gene expression distributions were displayed as violin plots with overlaid medians and quartiles. Volcano plots displayed effect sizes (log₂ fold change) against statistical significance (-log₁₀ p-values), with genes categorized as upregulated (log₂FC > 0.05, FDR < 0.05), downregulated (log₂FC < -0.05, FDR < 0.05), or not significant. Hierarchical clustering heatmaps were generated using z-score normalized expression values with color scaling (red = high expression, blue = low expression). Model performance was visualized through ROC curves, confusion matrices, feature importance plots, and cross-validation score distributions.

### Statistical analysis

All statistical analyses were performed in Python 3.10 using SciPy (v1.11.0), NumPy (v1.24.0), pandas (v2.0.0), and statsmodels (v0.14.0). The respective tests are mentioned for each analysis in the respective section. Effect sizes were quantified using Cohen’s d, calculated as the difference in means divided by the pooled standard deviation. Statistical significance was defined as p < 0.05 or FDR < 0.05 where applicable.

## Results

### Biological age analysis

#### DNA methylation status

**Fig. 2:**
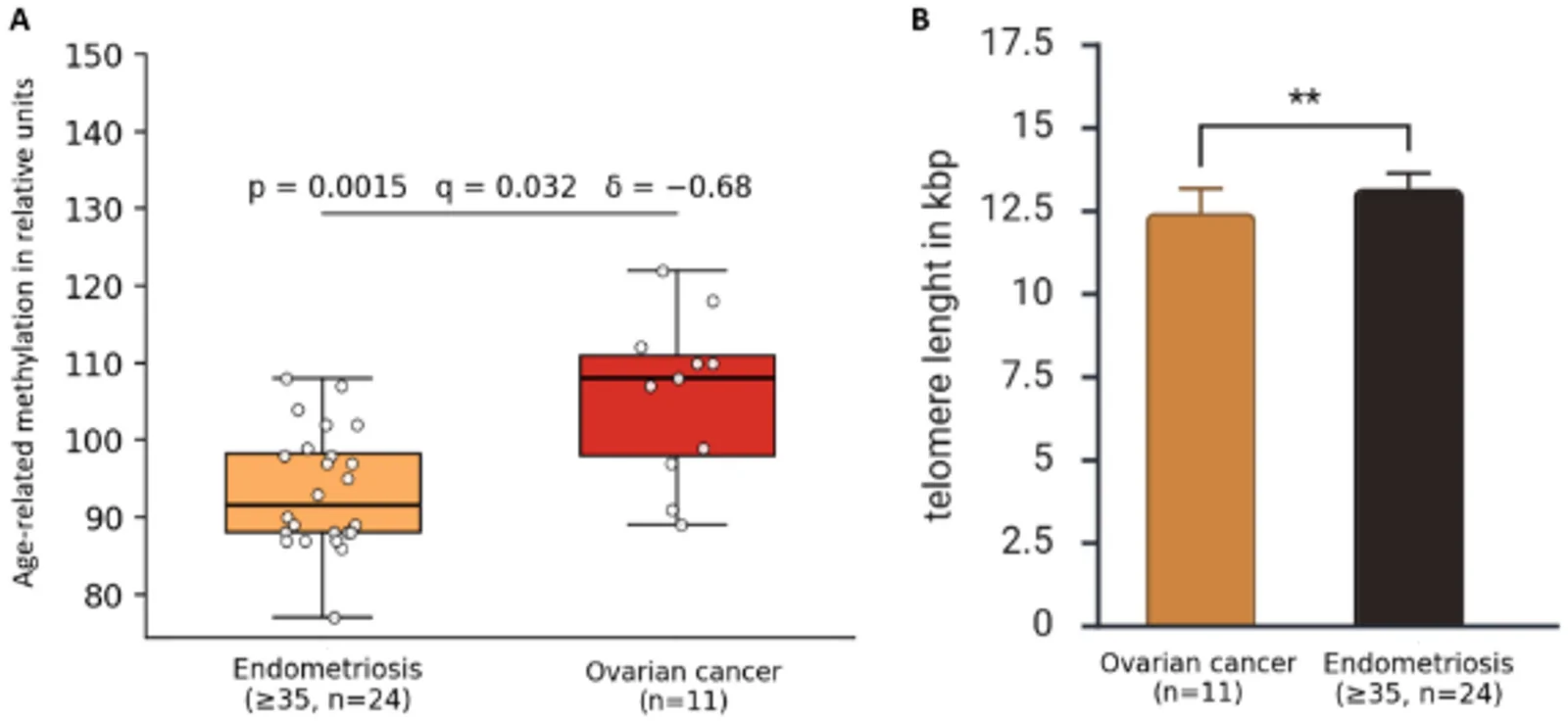
Age-matched comparison of age-related methylation and telomere length. (**A**) Boxplot showing age-related DNA methylation levels, expressed in relative units, in endometriosis samples from patients aged ≥ 35 years (*n* = 24) and EAOC samples (*n* = 11). EAOC patients exhibited significantly greater age-related methylation than endometriosis controls (*p* = 0.0015, q = 0.032, effect size δ = −0.68). Individual data points are overlaid on the boxplots. The center line indicates the median, boxes represent the interquartile range (IQR), and whiskers indicate the minimum and maximum values. (**B**) Comparison of telomere length between EAOC (*n* = 11) and endometriosis patients aged ≥ 35 years (*n* = 24). Telomere length is presented in kilobase pairs (kbp). Bars represent mean values with error bars indicating standard deviation (SD) with the indicated significance (**p < 0.01)

To assess the epigenetic landscape that undergoes predictable, stereotypical modifications during senescence, we analyzed divergent shifts in DNA methylation status and age-associated global hypomethylation, which is frequently observed within repetitive genomic elements such as LINE-1 sequences. Comparative analysis of age-related DNA methylation revealed a significant difference between EAOC and endometriosis patients. As shown in Fig. 2, age-matched individuals with endometriosis (≥ 35 years, *n* = 24) exhibited significantly lower (*p* = 0.0015) age-related methylation levels than EAOC patients (*n* = 11). This difference remained significant after multiple testing correction (*q* = 0.032). The effect size was large (Cliff’s δ = −0.68), indicating a substantial shift toward reduced methylation values in the endometriosis group (Fig. 2).

#### Telomere length

Due to the fact that telomeric sequences undergo progressive shortening with each successive mitotic division, the telomere length was evaluated in the respective study groups to analyze the so-called chromosomal clock, which regulates the replicative lifespan of cells and serves as a predictive indicator of biological aging trajectory [38]. Our telomere length analysis showed that patients with endometriosis had significantly (*p* = 0.00103) longer telomeres (13.18 ± 0.46 kbp) compared to EAOC patients (12.45 ± 0.72) (Fig. 2). This suggests differential cellular aging dynamics between the two patient groups.

#### Multi-metric comparison across analytical models

**Fig. 3:**
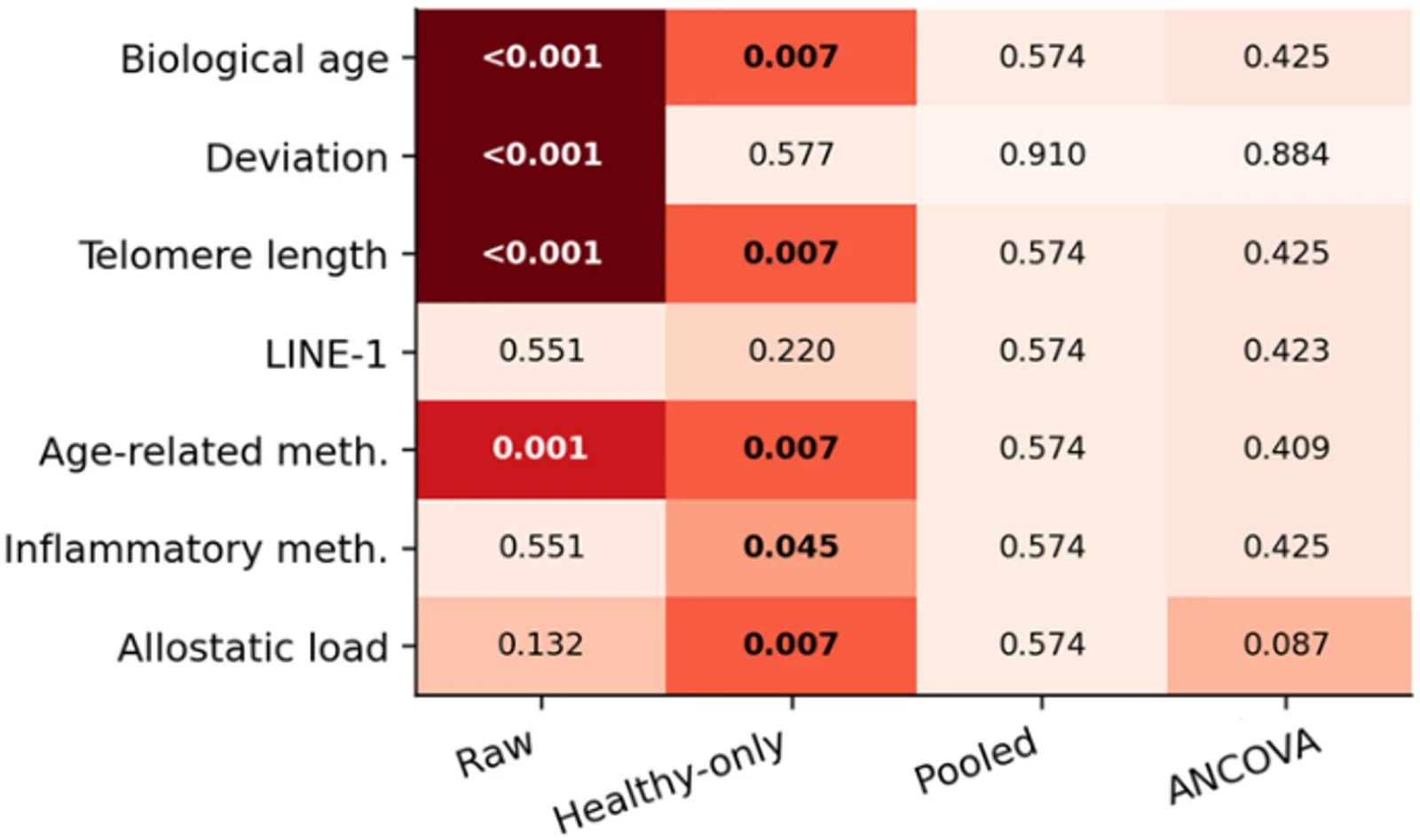
Three-group Kruskal–Wallis analysis of multiple aging-related biomarkers across adjustment methods. The bold numbers indicate significance (*p* < 0.05)

Long Interspersed Nuclear Element-1 (LINE-1) methylation serves as a robust surrogate marker for global DNA methylation homeostasis and overall genomic integrity. The progressive, age-associated attrition of methylation at these repetitive elements precipitates genomic instability and is clinically correlated with accelerated biological aging, heightened oxidative stress, and chronic systemic inflammation. Conversely, elevated LINE-1 methylation levels function as a protective mechanism, promoting genomic stability and potentially extending the physiological healthspan. As shown in the heatmap (Fig. 3), summarizing multiple aging-related biomarkers (biological age, age deviation, telomere length, LINE-1 methylation, age-related methylation, inflammatory methylation, and allostatic load) across different analytical models revealed no significant differences with regard to LINE-1 methylation. The biological age difference in the raw model is highly significant (*p* < 0.001) and remained significant in the healthy-only subset (*p* = 0.007). The deviation between biological age and chronological age was significant in raw data (*p* < 0.001), and was as follows: −14.64% years in the EAOC patients’ group, + 16.92% years in the endometriosis group, and + 26.87%. This showed that the EAOC patients had a biologically younger age than their chronological age. Moreover, telomere length was significantly different in raw (*p* < 0.001) and healthy-only analyses (*p* = 0.007), and age-related methylation was consistently significant in raw (*p* = 0.001) and healthy-only (*p* = 0.007) analyses. However, the inflammatory methylation status showed only modest significance in the healthy-only group (*p* = 0.045), whereas allostatic load was significant in the healthy-only subset (*p* = 0.007), but not in other models (Fig. 3). Notably, the cumulative physiological cost of chronic homeostatic maintenance is defined as allostatic load. While allostatic mechanisms initially facilitate adaptive stability in response to acute challenges, chronic exposure to stressors can overwhelm these regulatory frameworks, leading to long-term physiological deterioration and increased susceptibility to multi-systemic disease. Integrating these epigenetic markers with clinical data provides a more precise quantification of an individual’s biological aging trajectory compared to chronological age alone. The non-parametric comparisons (Mann–Whitney U test) confirmed that the age-related methylation was the only marker that remained statistically, strongly significant after false discovery rate correction (*p* = 0.0015; q = 0.032), with a large negative effect size (δ = −0.68). Other markers, including age deviation (*p* = 0.0122), biological age (*p* = 0.0528), telomere length (*p* = 0.0816), and allostatic load (*p* = 0.0955), showed nominal or borderline significance but did not withstand correction (q ≥ 0.128) (Table 1). However, LINE-1 methylation and inflammatory methylation were clearly non-significant (*p* > 0.3).

**Table 1:** Age-matched comparison (endometriosis ≥ 35 vs. ovarian cancer)

Marker	U	*p*	Cliff’s δ	q_BH
Age-related methylation	42.5	0.0015	-0.68	0.032
Deviation	203.0	0.0122	+ 0.54	0.128
Biological age	77.0	0.0528	-0.42	0.334
Telomere length	181.5	0.0816	+ 0.38	0.334
Allostatic load	176.5	0.0955	+ 0.34	0.334
LINE-1	156.5	0.3921	+ 0.19	0.856
Inflammatory methylation	124.0	0.7895	-0.06	0.856

### Differential gene expression

**Fig. 4:**
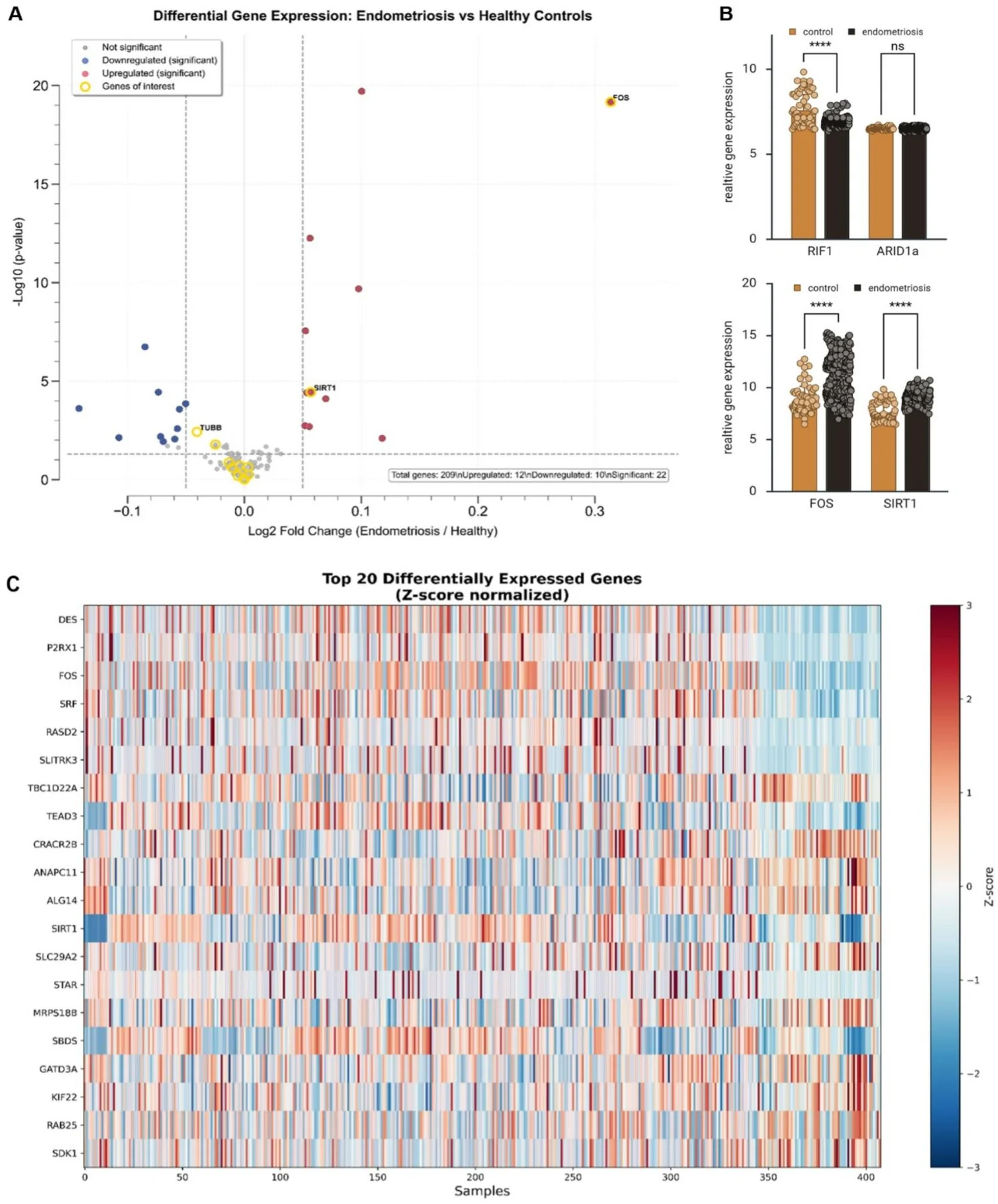
Differential gene expression in endometriosis patients and unaffected women. Panel **A** displays a volcano plot summarizing differential gene expression between endometriosis and healthy control samples, with log2 fold change on the x-axis and –log10(p-value) on the y-axis. A total of 22 genes reached statistical significance, including 12 downregulated and 10 upregulated in endometriosis. Panel **B** shows the box plots specifically highlighting the significant differences in gene expression for four genes of interest: *RIF1*,* ARID1A*,* FOS*, and *SIRT1.* Panel C shows a heatmap displaying the top 20 differentially expressed genes in patients with endometriosis compared to unaffected controls. Across all analyzed samples, with expression values Z-score normalized per gene. Each row represents a gene, and each column an individual sample. The color scale indicates relative expression, with red denoting higher-than-average expression and blue indicating lower-than-average expression. The intention was to preserve the predefined sample order and facilitate direct comparison between clinically defined groups, rather than perform unsupervised sample classification

To detect early changes in gene expression in endometriosis patients, we analyzed the EndoMet Database to identify early biomarkers for a potentially malignant transformation prior to the manifestation of EAOC. Most genes cluster around a log2 fold change of near zero, indicating relatively subtle but biologically relevant transcriptional alterations (Fig. 4). The genes *FOS*, *RIF1*, and *SIRT1* are significantly upregulated in endometriosis patients relative to controls (*p* < 0.0001). This illustrates that endometriosis is characterized by a distinct yet targeted transcriptional signature, rather than widespread gene expression remodeling. The significant upregulation of *RIF1*, *FOS*, and *SIRT1* suggests activation of pathways related to DNA damage response and replication control (*RIF1*), stress and immediate-early gene signaling (*FOS*), and metabolic regulation and epigenetic control of inflammation and aging (*SIRT1*). Overall, these results support a model in which endometriosis involves the coordinated dysregulation of genome maintenance, stress response, and metabolic pathways, contributing to disease persistence and progression.

As presented in the heatmap (Fig. 4), three genes, including *CRACR2B*, *TEAD3*, and *P2RX1*, show pronounced variability across samples, with clusters of elevated expression in subsets of individuals. Other genes, such as *DES*, *RASD2*, *SLITRK3*, and *KIF22*, display more moderate but consistent expression shifts. For example, stress- and signaling-related genes (*FOS*, *SRF*, *SIRT1*) tend to show concurrent increases in expression in overlapping sample groups, where as genes involved in structural or metabolic functions (*DES*, *STAR*, *MRPS18B*) exhibit more balanced or alternating expression states. The high proportion of differentially expressed genes appears to be due to the pathway-focused gene selection, biologically enriched target panels, and the potentially extensive molecular divergence associated with malignant transformation and altered biological aging trajectories

### Gene ontology enrichment analysis

**Fig. 5:**
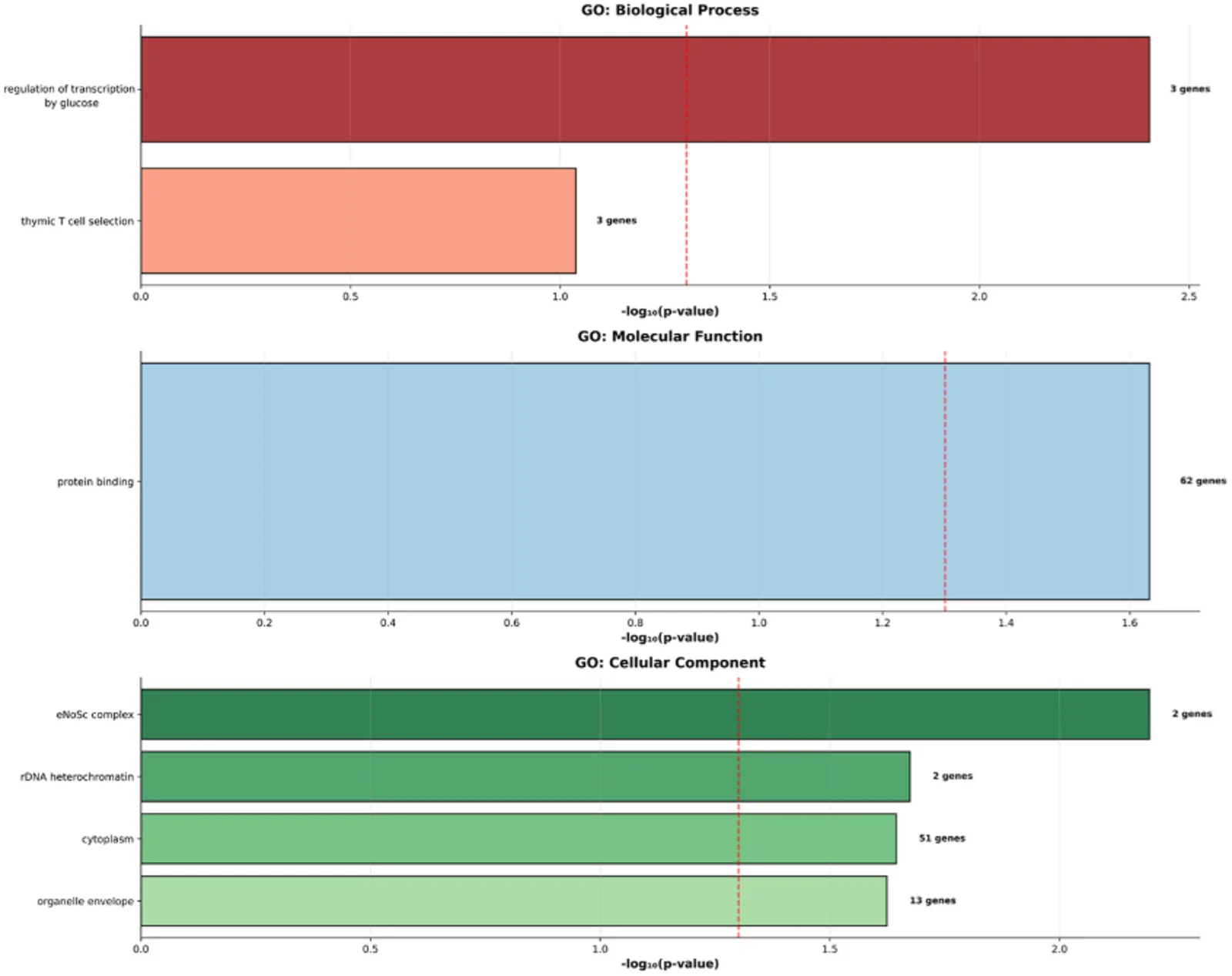
Gene Ontology (GO) enrichment analysis of genes associated with endometriosis. Three main GO domains are depicted (from top to bottom): Biological Process (BP), Molecular Function (MF), and Cellular Component (CC). Enrichment significance is shown as − log₁₀(p-value), with the red dashed line indicating the nominal significance threshold

A Gene Ontology (GO) enrichment analysis was performed to identify GO domains associated with genes related to endometriosis. As shown in Fig. 5, three main GO domains have been analyzed, such as Biological Process (BP), Molecular Function (MF), and Cellular Component (CC). Within the Biological Process category, the most significantly enriched term is regulation of transcription by glucose, exceeding the significance threshold and involving three genes. A second term, thymic T cell selection, showed a weaker enrichment and did not reach the indicated significance threshold, also comprising three genes. In the Molecular Function domain, protein binding emerged as the dominant enriched term, encompassing a large number of genes (*n* = 52). Although the enrichment approached the significance threshold, it reflects a broad functional category rather than a highly specific molecular activity. The Cellular Component analysis identified several enriched compartments. The eNOS complex and rDNA heterochromatin showed a strong enrichment despite involving a small number of genes (two genes each). More general cellular localizations, including cytoplasm (51 genes) and organelle envelope (13 genes), were also enriched and approached or surpassed the significance threshold.

### Machine learning models as prediction analysis

**Fig. 6:**
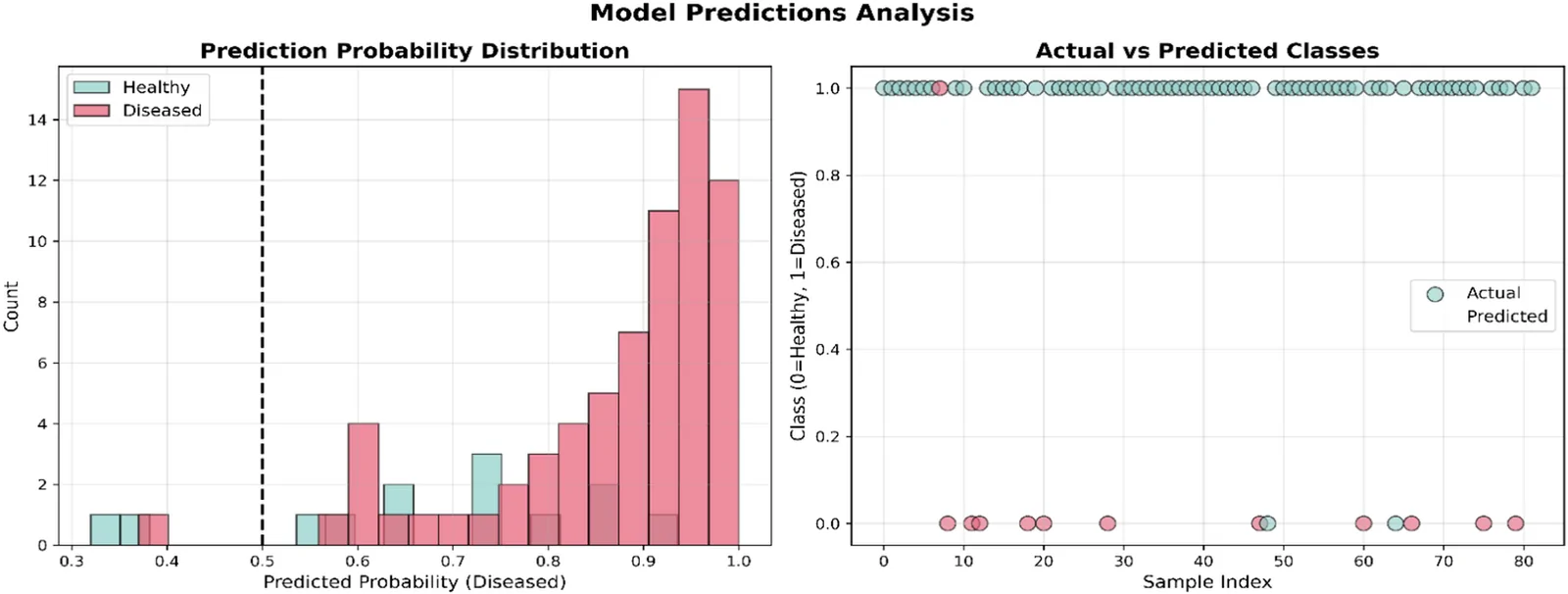
Analysis of the classification model’s prediction behavior of differentially expressed genes. In the left panel, the distribution of predicted probabilities for the diseased class is shown for healthy and diseased samples. The dashed vertical line at a probability threshold of 0.5 indicates the decision boundary used for classification, which clearly separates the majority of healthy and diseased samples. In the right panel, actual versus predicted class assignments are shown across all samples

**Fig. 7:**
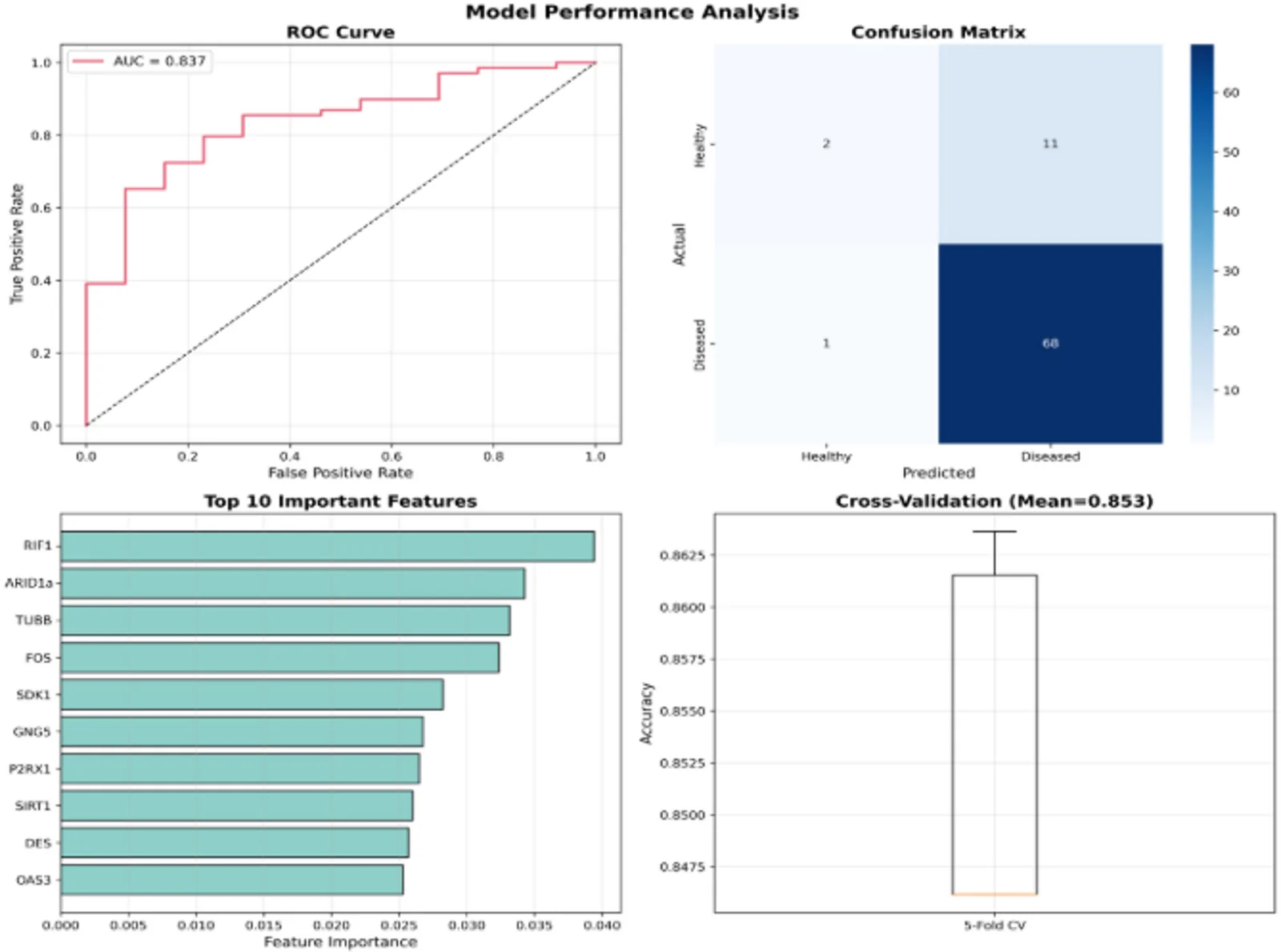
Performance and interpretability of the classification model across multiple evaluation metrics. The ROC curve (top left) demonstrates strong discriminative ability, with an area under the curve (AUC) of 0.837. The confusion matrix (top right) shows that the model correctly classifies the majority of samples. The feature importance analysis (bottom left) highlights the top ten predictors contributing to the model’s decision-making. The cross-validation results (bottom right)

To assess the utility of differentially expressed genes for classifying endometriosis samples, we constructed machine learning models using the top 50 most significantly differentially expressed genes as features. Endometriosis samples cluster predominantly at high predicted probabilities (approximately 0.8–1.0), indicating strong model confidence in identifying affected cases (Fig. 6). Control samples from unaffected women are primarily distributed at lower probability values, although a small overlap with predictions for diseased individuals is observed. Most samples are correctly classified, with predicted classes closely matching the true labels. Only a limited number of misclassifications are observed, appearing as discrepancies between actual and predicted class points, indicating isolated false-positive or false-negative predictions (Fig. 6). The actual versus predicted class comparison further supports the robustness of the model, showing a high overall classification accuracy with only a few errors. These misclassifications likely reflect biological heterogeneity or borderline cases rather than systematic model bias. Overall, these results indicate that the model reliably distinguishes healthy from diseased samples and provides well-calibrated probability estimates, supporting its potential utility for diagnostic or risk stratification applications. The ROC curve in Fig. 7 demonstrates a strong discriminative ability, with an area under the curve (AUC) of 0.837, indicating that the model effectively distinguishes between healthy and diseased samples across a range of classification thresholds. Specifically, 68 diseased cases and two healthy cases are correctly identified, while 11 healthy samples are misclassified as diseased (false positives) and one diseased sample is misclassified as healthy (false negative) (Fig. 7). This indicates high sensitivity for detecting disease, with a relatively low number of missed cases. Furthermore, we have performed a feature importance analysis, highlighting the top 10 predictors contributing to the model’s decision-making. The top differentially expressed genes included *RIF1* (log₂FC = 0.33, *p* = 8.79 × 10⁻²⁵), *ARID1a* (log₂FC = -0.10, *p* = 2.01 × 10⁻²⁰), *FOS (*log₂FC = 0.30, *p* = 1.57 × 10⁻¹⁹), and *TUBB* (log₂FC = 0.08, *p* = 7.18 × 10⁻¹⁰). These results suggest that genes involved in cytoskeletal organization, transcriptional regulation, and stress response play key roles in distinguishing between disease statuses. The cross-validation results demonstrate robust, stable model performance, with a mean accuracy of 0.853 across five folds and a narrow distribution, indicating low variability (Fig. 7).

## Discussion

Our study revealed, to our best knowledge, for the first time, a profound divergence in biological aging trajectories between patients suffering from endometriosis and those with endometriosis-associated ovarian cancer. Here, we provide novel and compelling insights into the distinct biological characteristics of DNA methylation status, biological age, longevity markers, and telomeres in endometriosis-associated ovarian cancer, underscoring their potential as molecular biomarkers for early detection at the stage of endometriosis. Specifically, while endometriosis patients exhibit significant epigenetic age acceleration and longer telomeres, EAOC patients exhibit a biologically younger profile and shorter telomeres.

Our observation that EAOC patients exhibit a biological age approximately 14.64% younger than their chronological age represents a significant finding. In contrast, endometriosis patients showed an age acceleration of + 16.92%. This discrepancy may be explained by the epigenetic rejuvenation phenomenon observed in certain malignancies. Research has shown that while cancer is generally associated with epigenetic age acceleration in peripheral blood, the tumors themselves, and sometimes the systemic environment, can capture the epigenetic signature of the tumor-initiating cell, which may possess a more primitive or stem-like DNA methylation state [39].

The interpretation of apparent epigenetic age reduction within the context of cancer biology requires a nuanced discussion that distinguishes between true physiological rejuvenation and the aberrant reprogramming characteristic of malignant transformation. While conventional epigenetic clocks typically measure biological age, the observation of a younger epigenetic signature in our study was based on a peripheral blood-based analysis in EAOC patients, which often reflects a departure from normal aging trajectories toward primitive, stem-cell-like states. Consequently, rather than representing an anti-aging process, apparent epigenetic de-aging often reflects tumor-associated dedifferentiation [40]. This phenomenon is driven by the acquisition of plastic, aberrant cancer stem-cell states where tissue-specific transcriptional factors are irreversibly suppressed, increasing both epigenetic and transcriptomic entropy [40]. The concept of methylation entropy is central to understanding this mechanistic distinction. DNA methylation drift, characterized by the accumulation of stochastic errors during replication, is intrinsically linked to stem cell division rates [41]. In cancer, this entropy increases, leading to a hybrid state of aberrant epigenetic flexibility where the distinction between stable cell fates is lost [42]. Noteworthy, in aging cells, methylation errors accumulate and lead to epiallelic shifts that predispose tissues to malignancy, even in younger individuals [41]. The reduction in epigenetic age observed in aggressive tumors may also result from clonal selection for highly proliferative cell populations. Increased proliferation inherently links methylation drift with age to the rate of stem cell turnover [41]. Previous research has shown that deregulation of histone modifications creates a permissive chromatin environment that supports tumor growth and survival rather than physiological health [43].

Furthermore, recent studies using pan-tissue clocks have demonstrated that while eutopic endometrium remains concordant with chronological age, ectopic lesions often exhibit significant age deceleration, appearing up to 16 years younger than chronological age [44]. This suggests that the malignant progression from endometriosis to EAOC may involve a fundamental reprogramming of the epigenetic clock towards a less mature developmental state. The significantly longer telomeres observed in endometriosis patients compared to EAOC patients is a very interesting finding. High telomerase activity and human telomerase reverse transcriptase (hTERT) levels have been documented in the eutopic and ectopic endometria of women with endometriosis, facilitating the survival and proliferation of these cells in ectopic environments [45]. This telomeric robustness may be necessary for the persistence of benign lesions [46]. Conversely, the shorter telomeres in EAOC patients reflect the exhaustive mitotic history and rapid cellular turnover characteristic of malignant cells [47]. The transition from a benign state (with telomere lengthening) to a malignant state (with telomeric attrition) represents a critical shift in the cellular aging dynamics of the ovarian niche [48].

The most robust marker in our analysis was age-related methylation, which remained highly significant even after multiple testing correction. The lower methylation levels in endometriosis patients suggest a more pronounced shift toward global hypomethylation compared to EAOC. While global hypomethylation, specifically of LINE-1 elements, is a classic hallmark of both aging and cancer that leads to genomic instability, our results showed no significant difference in LINE-1 methylation between the two groups. This suggests that the epigenetic differences between endometriosis and EAOC are driven by specific CpG-rich regional modifications rather than a systemic collapse of global methylation stability [49].

Moreover, our findings are particularly significant given that *RIF1* overexpression has been consistently linked to aggressive disease, worse overall survival, and increased chemoresistance in epithelial ovarian cancer generally [50, 51]. The known roles of *RIF1* in DNA replication regulation and DNA repair pathways suggest that its dysregulation could contribute to genomic instability characteristic of cancer progression [52, 53]. The observed expression pattern of *RIF1* in our study, therefore, implicates it not only as a potential prognostic indicator but also as a marker reflecting the underlying mechanisms of tumor maintenance or resistance, warranting further investigation in the context of EAOC progression. The performed machine learning models and prediction analysis achieved a robust performance (AUC = 0.837, mean accuracy = 0.853), effectively discriminating between healthy and diseased states. The high AUC and cross-validation accuracy support the model’s ability to generalize beyond the training data. Our findings suggest that the model is both predictive and mechanistically informative, making it a promising tool for disease classification and uncovering molecular signatures associated with disease status. Notably, *FOS* overexpression has previously been shown to enhance the malignant potential of eutopic endometrial stromal cells in EAOC patients [50, 54], and *TUBB* has been identified as a prognostic marker for EAOC [55]. These findings validate the biological relevance of our model’s key features and suggest their utility in a multi-marker diagnostic or prognostic panel.

The Gene Ontology enrichment analysis further elucidated the underlying biological processes in endometriosis, indicating a significant involvement of metabolic and transcriptional regulation, as well as immune-related processes. The enrichment of transcription regulation by glucose aligns with emerging evidence of metabolic reprogramming in endometriotic and ovarian cancer cells, including a preference for aerobic glycolysis [56]. The presence of thymic T cell selection and protein binding as dominant molecular functions supports the known inflammatory nature of endometriosis, which is relevant to the complex immune microenvironment of ovarian cancer [56–59]. Enrichment in cellular components such as the eNOS complex and rDNA heterochromatin suggests roles for nitric oxide signaling and chromatin organization, further emphasizing the multifaceted cellular dysregulation in this disease. Interestingly, the observed significant telomere shortening in EAOC patients is consistent with increased cellular turnover, genomic instability, and replicative stress associated with tumorigenesis [60]. In contrast, patients with endometriosis exhibit comparatively longer telomeres, suggesting a less advanced state of cellular aging and genomic damage. The observed difference between the groups highlights a potential role of telomere dynamics in the progression from benign endometriotic lesions to malignant transformation [61].

## Conclusions

In conclusion, our study offers a new perspective, emphasizing that EAOC is a distinct clinical and molecular entity and identifies key molecular players that are also characteristic of aging. The robust performance of our classification model, coupled with biologically meaningful gene enrichment, underscores the potential for developing multi-marker panels for improved diagnostic accuracy and prognostic stratification. The limitations of this study primarily stem from its retrospective design and the low number of available patient samples. Future prospective studies with larger cohorts are warranted to validate these markers and integrate them into clinical practice for earlier detection and personalized management strategies for EAOC. The absence of an independent external validation cohort limits the robustness and generalizability of the findings. Moreover, the biological aging analyses were predominantly based on peripheral blood-derived biomarkers and epigenetic age estimates. Consequently, it remains uncertain to what extent the observed aging signatures reflect tumor-intrinsic biological processes within endometriosis-associated ovarian cancer or, alternatively, systemic host responses. Blood-derived epigenetic clocks may be influenced by multiple factors, including variations in immune-cell composition, chronic inflammatory status, endocrine alterations, and prior therapeutic interventions. Although peripheral blood biomarkers offer important translational advantages due to their accessibility and clinical applicability, their tissue specificity remains limited.

## Supplementary Information

Below is the link to the electronic supplementary material.

Supplementary Material 1

## Data Availability

Patient-related data and data regarding the biological age will be made available upon request. Data regarding gene expression in women with endometriosis can be found at the Turku Endometriosis Database.

## Abbreviations

Abbreviation: Explanation
ANCOVA: Analysis of Covariance
AUC: Area Under the Receiver Operating Characteristic Curve
BP: Biological Process (Gene Ontology category)
CA: Chronological Age
CC: Cellular Component (Gene Ontology category)
CI: Confidence Interval
CV: Coefficient of Variation
DAVID: Database for Annotation, Visualization and Integrated Discovery
DES: Deep Endometriosis
DNA: Deoxyribonucleic Acid
EAOC: Endometriosis-Associated Ovarian Cancer
FDR: False Discovery Rate
FIGO: International Federation of Gynecology and Obstetrics
GO: Gene Ontology
IQR: Interquartile Range
LINE-1: Long Interspersed Nuclear Element-1
MA: Methylation Age
MF: Molecular Function (Gene Ontology category)
MSD: Meso Scale Discovery
MWU: Mann–Whitney U Test
RNA: Ribonucleic Acid
ROC: Receiver Operating Characteristic
SD: Standard Deviation
