# Supplementary material for: Multimodal analysis reveals asynchronous aging dynamics between endometriosis-associated ovarian cancer and endometriosis patients
Source: BMC Med. 2026 Jul 27;24:589. doi: 10.1186/s12916-026-05101-6 (PMC13700490; doi:10.1186/s12916-026-05101-6)
Supplement: Supplementary file 1 [file 12916_2026_5101_MOESM1_ESM.docx]

**Supplementary File 1**

**List of 215 analyzed genes**

*TUBB*

*FOS*

*TERT*

*BRCA1*

*BRCA2*

*SIRT1*

*DNMT1*

*DNMT3A*

*DNMT3A*

*DNMT3B*

*ARID1A*

*RIF1*

*TP53RK*

*KRAS*

*TP53TG1*

*RERE*

*LOC105374121*

*CIDEA*

*SLC17A3*

*ATP6V1C2*

*ZNF768*

*F3*

*HTR5BP*

*LINC00999*

*GAK*

*COMMD4*

*LIN52*

*LOC102724135*

*PCOTH*

*FXN*

*CD164*

*MOB3A*

*OR1J2*

*GNG5*

*EXTL3*

*CNPY3*

*FAM174C*

*LOC154449*

*GAS2L2*

*GATD3A*

*URGCP*

*NF1P1*

*DNAH14*

*OR8H1*

*ANAPC11*

*METRNL*

*ARHGAP24*

*P2RX1*

*MTPN*

*LRR1*

*UBLCP1*

*TAF5L*

*ADAMTSL4*

*ZPBP2*

*DES*

*HERC2P9*

*KIF22*

*HIPK2*

*SHMT2*

*MRPS5*

*SPN*

*OAS3*

*CHRNA6*

*TRPC4*

*TBC1D22A*

*RPL35P1*

*ST6GALNAC4*

*TM2D1*

*LRRC23*

*RASD2*

*PTTG1*

*UBE2DNL*

*ITGA10*

*ETFRF1*

*PLPPR1*

*MARS2*

*UTY*

*MAGEC1*

*EAPP*

*PDIA6*

*NFATC3*

*CYP2B6*

*FGFBP2*

*HBS1L*

*LRRC23*

*RBMY1E*

*SDK1*

*PNCK*

*NRDE2*

*SLC9B1P1*

*CRACR2B*

*REEP3*

*ZNF365*

*BTBD3*

*RBM17*

*LINC01140*

*TRNAU1AP*

*IL21R*

*SYNE2*

*RASSF4*

*GPR173*

*OR8H2*

*SLC4A1*

*ANTXR2*

*MIEN1*

*TBXT*

*CENPV*

*TMEM59*

*RHOXF2*

*TXNDC11*

*DOCK2*

*LRRC2*

*LINC00607*

*PDXDC2P*

*SRF*

*LOC105369958*

*SLITRK3*

*PSEN2*

*KRT8P47*

*LLPHP3*

*ATG10*

*TPT1*

*OGDH*

*OTUD1*

*NDUFAF3*

*GABARAPL2*

*ADAMTS6*

*CREB3L2*

*PANX3*

*LINC00472*

*CD52*

*ASB16-AS1*

*H2AC16*

*SLC6A17*

*ARPC5*

*GABRA2*

*AFAP1L1*

*OPN5*

*CASC15*

*F11*

*DHX35*

*SUV39H1*

*REPS2*

*SLC7A1*

*PRDM4*

*TMTC3*

*SEPTIN11*

*HAUS3*

*NR2C2*

*NOXO1*

*DPY19L2P2*

*MKI67*

*CASP8*

*FLJ16779*

*CCDC179*

*RNF144A*

*BHMT2*

*CHST6*

*GRASP*

*UVSSA*

*BRF2*

*LOC107986637*

*MASTL*

*PRR30*

*CLDN3*

*SPRTN*

*LYZL4*

*ZNF700*

*SLC29A2*

*TDRD12*

*C1orf194*

*SIX4*

*ZNF613*

*BSG*

*LOC105379332*

*HINT3*

*PDCD10*

*TEAD3*

*FOXN3-AS1*

*STAR*

*REP15*

*CDH16*

*LOC101927531*

*LOC100129603*

*CLNK*

*TMEM65*

*FOXP4*

*MRPS18B*

*VN1R1*

*ANKRD16*

*TEKT5*

*METRNL*

*FSCN2*

*LINC01210*

*DIPK1B*

*ALG14*

*FHOD1*

*CARF*

*ADGRG7*

*MORC2*

*GRHL3*

*SBDS*

*ANO2*

*ZNF516-DT*

*KIF25*

*ACKR1*

*ZNF318*

*RAB25*

*FAM83B*

*LRRC8C*

*DPPA4*

*GAGE2E*

*DFFA*

*NDUFA3*

*KLLN*
